# Edible cannabis for chronic low back pain: associations with pain, mood, and intoxication

**DOI:** 10.3389/fphar.2024.1464005

**Published:** 2024-09-24

**Authors:** Samantha N. Melendez, Marco Ortiz Torres, Jonathan K. Lisano, Gregory Giordano, Carillon Skrzynski, Kent E. Hutchison, Angela D. Bryan, L. Cinnamon Bidwell

**Affiliations:** ^1^ Department of Psychology and Neuroscience, College of Arts and Sciences, University of Colorado Boulder, Boulder, CO, United States; ^2^ Institute of Cognitive Science, University of Colorado Boulder, Boulder, CO, United States; ^3^ Department of Psychiatry, School of Medicine, University of Colorado Anschutz Medical Campus, Denver, CO, United States

**Keywords:** cannabinoids, cannabis, edibles, chronic pain, observational, THC, CBD, side effects

## Abstract

**Introduction:**

Cannabis, commonly known for both therapeutic and intoxicating effects, is gaining accessibility on legal markets and traction as a potential alternative therapy for pain mediation, particularly in those suffering from chronic low back pain. However, the effectiveness in this population of legal market forms of cannabis, particularly commonly used edibles, is unknown.

**Methods:**

Therefore, this study utilized a naturalistic prospective design where participants with chronic low back pain with intentions to initiate cannabis use for treatment were recruited and self-selected edible cannabis products containing varying amounts of delta- 9 tetrahydrocannabinol (THC) and cannabidiol (CBD). Products were categorized as CBD-dominant, THC-dominant, or combined THC and CBD (THC + CBD).

**Results:**

249 participants [140 female (56.62%), mean (SD) age of 46.30 (16.02), 90% White] were tracked over 2 weeks of *ad libitum* use and assessed during a naturalistic acute cannabis administration session on changes in pain, mood, and subjective drug effects. During acute administration, a significant correlation between THC dose and short-term pain relief was found, suggesting that higher THC doses were associated with greater pain reduction (*p* < .05). In addition, THC was associated with higher levels of subjective cannabis drug effects (*p* < .001), regardless of whether CBD was also in the edible product. Acute CBD dose was primarily associated with short-term tension relief (*p* < .05); however, there were no associations between CBD dose and acute pain. Over the 2-week ad libitum administration period results suggested pain reductions across participants using all forms of cannabis. However, trends suggested that more frequent use of CBD-dominant edible cannabis may be associated with greater reductions in perceived pain over the 2-week observation period (*p* = .07).

**Discussion:**

These findings support the short-term analgesic effects of THC and anxiolytic effects of CBD and further suggest that orally-administered THC and CBD should continue to be evaluated for the potential to provide both acute and extended relief from chronic low back pain.

**Clinical Trial Registration:**

https://clinicaltrials.gov/study/NCT03522324?locStr=Boulder,%20CO&country=United%20States&state=Colorado&city=Boulder&cond=chronic%20low%20back%20pain&intr=Cannabis&rank=1, identifier NCT03522324.

## Introduction

It is estimated that chronic pain (pain lasting longer than 12 weeks) impacts approximately 76 million Americans every year (Medicine AAoP). A major issue in the management of chronic pain is a lack of consistent, effective treatments. Due to the ubiquity of chronic pain, an increasing number of patients are turning to alternative pain therapies, like cannabis. Self-report data indicates that 87%–94% of medical cannabis patients are using cannabis for pain relief (Light et al., 2014; Ilgen et al., 2013). Further, evidence suggests that some individuals are using cannabis to supplement their traditional pain medications. A survey study reported that the use of medical cannabis in pain patients was associated with a 64% decrease in prescribed opiate dosage (Boehnke et al., 2016). While cannabis is not an FDA-approved medicine for chronic pain, these reports indicate there is a strong need for research into the efficacy and mechanisms underlying the effects of cannabis on pain.

There is pre-clinical evidence that suggests hemp and cannabis oil is effective in reducing hypersensitivity for neuropathic pain (Linher-Melville et al., 2020; Vigil et al., 2020). Several human studies on cannabis and pain utilizing a large-scale naturalistic dataset have found that patients engaging in self-directed use of cannabis products (e.g., oils, pills, edibles, and smokables) reported a significant average pain reduction and pain symptom relief (Li et al., 2019; Stith et al., 2018; Stith et al., 2019). However, the majority of human clinical trials on cannabinoids and pain have evaluated nabiximols (cannabis-based oral spray) or cannabis flower that was vaporized or smoked which have been found to reduce pain (Whiting et al., 2015a; Safakish et al., 2020; Cuttler et al., 2022). Although this research is promising, few studies have evaluated edible cannabis products for pain which are prevalent in legal markets and accessible to patients. In fact, data suggests that pain patients are more likely to use edible products relative to other forms of use (Colorado Department of Revenue, 2014). Thus, while the use of orally sprayed and inhaled cannabis for the treatment of pain has been supported, little is known about the efficacy, dose, short-term (acute), and long-term (extended) effects of cannabis edible products for pain.

Cannabinoid physiology and pharmacology is complex (Laaboudi et al., 2024), however, the existing literature on the pharmacodynamics of edible cannabis have reported that the effects of cannabis are delayed when compared to inhalation methods (Schlienz et al., 2020). For edible products, subjective effects are perceptible at 30 min and peak around 1.5–3 h post-ingestion while cognitive impairment peaks around 2–5 h. Limited pharmacokinetic research on edible cannabis has shown great variability and discrepancies in outcomes, similar to that of smoked cannabis. However, it has been reported that THC peak concentration (C_max_) ranged between 2.5 ng/mL to 5.5 ng/mL with the time range variation (T_max_) between 35 min and 2 h for ingested cannabis (Ewell et al., 2021; Vandrey et al., 2017).

Altogether, the two most studied cannabinoids, 9-delta-tetrahydrocannabinol (THC) and cannabidiol (CBD), are heavily implicated in the analgesic properties of cannabis (Andreae et al., 2015; Richards et al., 2012; Kulesza et al., 2024; Cohen et al., 2019). Emerging research even suggests that the two may work better synergistically than individually (Pennypacker and Romero Sandoval, 2020; Freeman et al., 2019; Wall et al., 2022). However, it is well known that THC is associated with intoxication and cognitive effects (e.g., mild euphoria, relaxation, sensory alterations, immediate and delayed recall, and reduced working memory) (Abel, 1979; Darley et al., 1974) which may be a deterrent to prospective patients. Therefore, it is imperative to assess any negative cannabis effects on mood and intoxication concurrently with its effects on pain to better understand the benefits and harms of cannabis. Considering the many challenges associated with assessing participants in a real-world setting following cannabis use, our research team utilizes an innovative strategy via our mobile pharmacology laboratory. This mobile laboratory, which is equipped to conduct assessments, collect blood, and take vital signs, is parked outside of the participants residence in order to obtain real-time, in-person data collection prior to and after cannabis consumption.

The present study used a prospective design to examine the use of edible cannabis products containing a spectrum of THC and CBD among individuals with chronic low back pain. The primary aim of this study was to observe pain and intoxication under the acute naturalistic use of edible cannabis and assess if changes in these outcomes were associated with products higher or lower in THC and CBD. Based on the evidence of acute THC-induced analgesia, we hypothesized that edible products containing higher amounts of THC would be most effective in acute pain relief, but would also be associated with negative effects on mood and intoxication (Weizman et al., 2018; Elikkottil et al., 2009; Rabgay et al., 2020). Our secondary aim was to assess if the use of *ad libitum* cannabis over an extended 2-week period was implicated in changes in perceived pain and if these changes were more associated with THC or CBD. Similar to the acute effects, we believed higher doses of THC over the extended 2-week period would be associated with reductions in pain. However, due to previous observations of CBD reducing negative effects of high-dose THC (Sharpe et al., 2020; Solowij et al., 2019), we predicted that products with relatively equal amounts of THC and CBD would be associated with pain reduction and lower intoxication, which may help determine the most beneficial cannabinoid profile for chronic pain patients.

## Methods

### Participants

**FIGURE 1:**
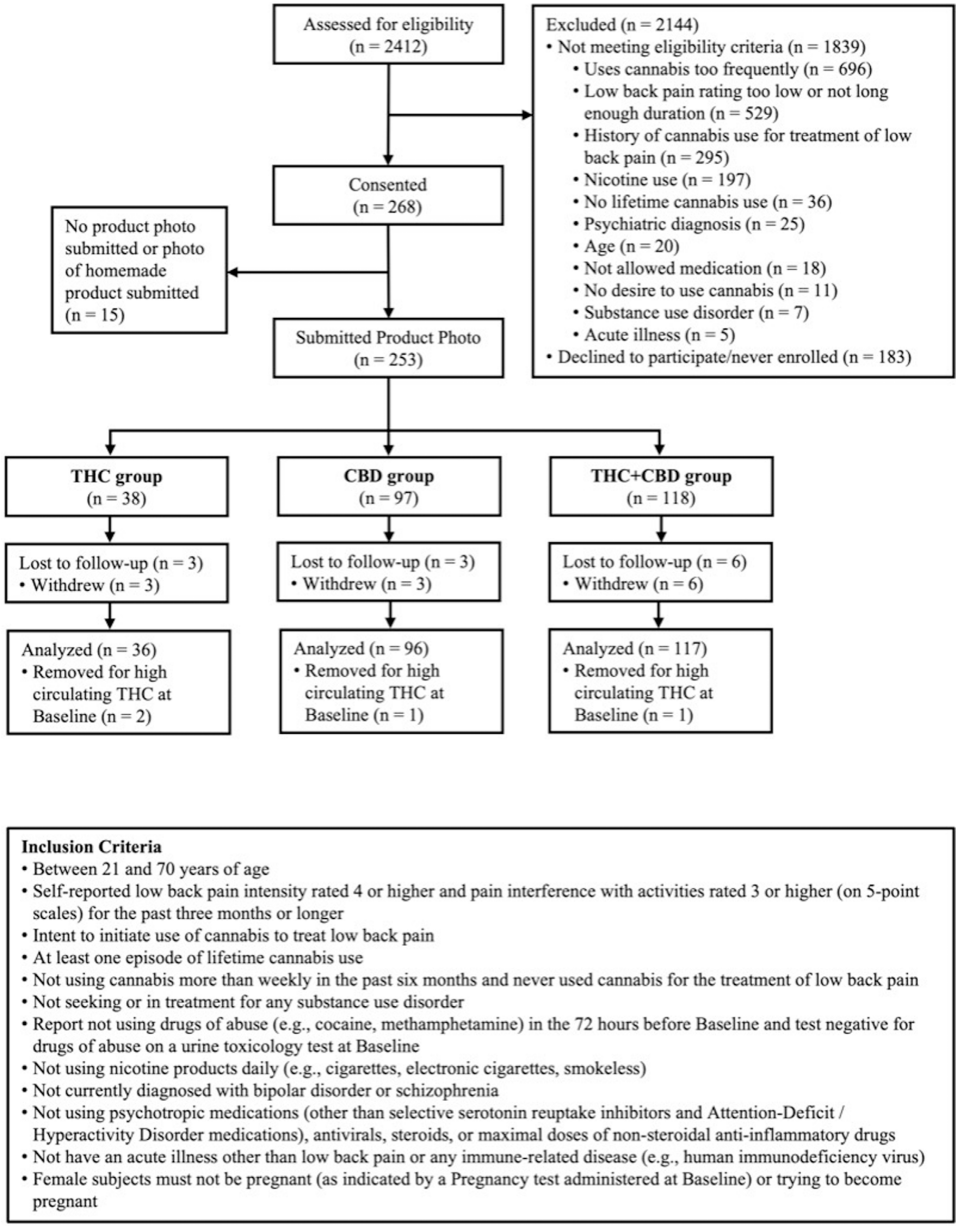
Participant Consort and Inclusion Criteria. Figure 1 is a flow diagram of participant eligibility assessment, enrollment, product selection, and analysis. Subjects recruited for the study were assessed and those eligible were consented and enrolled. Participants self-selected an edible cannabis product and were grouped by THC, CBD, or THC + CBD.

Beginning in June of 2018 and ending in April of 2023, participants were recruited through community events, social media, and mailed and posted flyers in the Denver-Boulder metro area. Participants were deemed eligible if they reported ≥3 months of low back pain, with pain intensity greater than four and pain interference with activities greater than three measured on 1–5-point scales (Broderick et al., 2013). Further, to be eligible participants must have intended to initiate cannabis use for the treatment of their low back pain and could not be cannabis naive (had to have at least one previous lifetime use of cannabis) but could not be using cannabis more than weekly for the past 6 months. A full list of inclusion and exclusion criteria is provided in Figure 1. This study was preregistered at Clinicaltrials.gov (NCT03522324), followed all ethical standards for the 2008 revision of the Helsinki Declaration, and was approved by the Institutional Review Board of the University of Colorado Boulder.

### Timeline and Compensation

After participants were screened for inclusion (Figure 1), eligible participants were scheduled for two in-person study visits over 2 weeks. The first study visit (Baseline) was conducted at our on-campus laboratory, and the second visit (Week 2) was conducted in our mobile pharmacology laboratory at the participant’s place of residence. Participants were compensated $220 for the completion of all study visits.

#### Baseline visit

**FIGURE 2:**
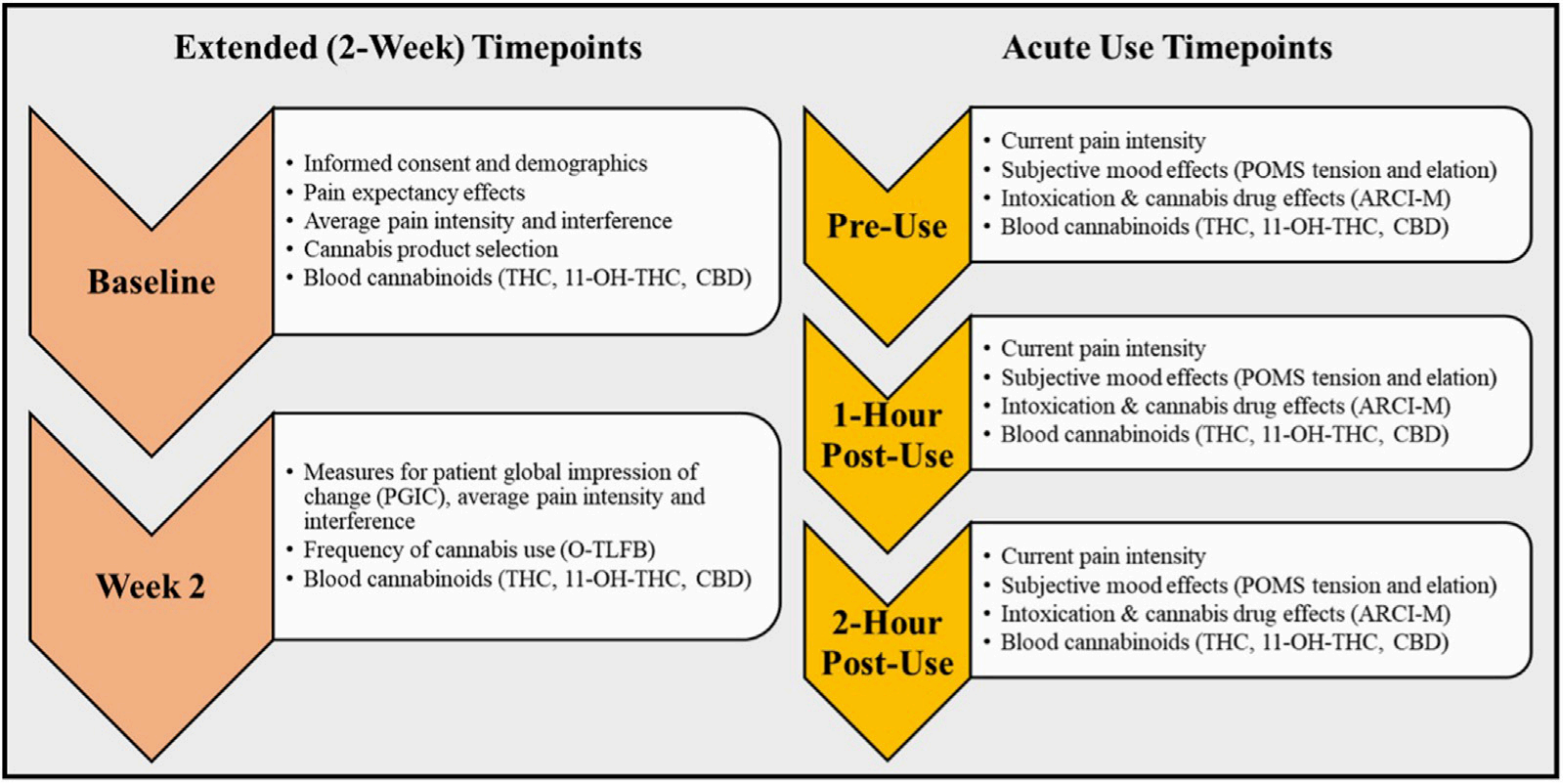
Study Timepoints and Outcomes for Acute Use and Two-week Observation Periods. This diagram details the participant study visits over the Two-week and at the Acute Use timepoints, including the actives and assessments completed at each visit.

To initiate the Baseline visit, participant eligibility was verified and informed consent was obtained. During this visit, participants completed various questionnaires to assess the primary and secondary outcomes of the study (Figure 2). In addition, participants provided medical history and demographic information.

##### Cannabis product selection and use procedures

At the end of the Baseline visit, a research assistant provided participants with information regarding the range of edible cannabis products available, prices, and nearby locations where the products could be purchased. Participants were given a brief safety orientation about edible cannabis use based on the state of Colorado’s public health materials (e.g., “start low, go slow”). Participants were then instructed to purchase enough edible product, from any local dispensary, to fit their *ad libitum* use over the next 2 weeks. In addition, participants were instructed to refrain from using any cannabis product other than their purchased study product during the study observation. The local dispensaries had no role in the study design, data collection, analysis, interpretation, writing of the report, or funding of this project.

It is important to note that many individuals with medical motives for cannabis use will initiate their use through the recreational market. Only if the products effectively address their symptoms, will they then pursue a medical card to purchase cannabis products through medical dispensaries. As specified by the state of Colorado, all cannabis products sold at dispensaries must be labeled with THC and CBD content following testing in an International Organization of Standards 17,025 accredited laboratory. This labeling was used to verify product selection via participants uploading a photo of their purchased product label through RedCap (Harris et al., 2009), a secure data gathering system. Product label photos were used to record milligrams of THC and CBD per serving and to determine the THC:CBD ratio for cannabinoid group stratification described below. Due to the state labeling requirements, participants were not blinded to the product that they were using; however, research personnel who interacted with participants remained blind until the conclusion of data collection.

##### Strain stratification

Consistent with the wide range of cannabis formulations on the legal market, the self-selected products contained various ratios of THC and CBD. The selected products were collapsed into the following categories: CBD-dominant products had more than five times CBD compared to THC (e.g., 25 mg CBD, 0 mg THC); THC-dominant products had more than five times THC compared to CBD (e.g., 25 mg THC, 0 mg CBD); and all products that did not meet the five times threshold to be classified as either CBD- or THC-dominant were categorized as THC + CBD.

#### Acute administration and mobile laboratory visit

The following methodology adheres to federal cannabis guidelines and is compliant with the Drug-Free Schools Act. The Week two visit was completed in our mobile pharmacology laboratory which was driven to and parked outside of the participant’s residence. Before using any cannabis that day (pre-use), participants completed a blood draw for evaluation of cannabinoid biomarkers and reassessment for pain intensity, pain interference, and frequency of cannabis and alcohol use (Figure 2). Participants were then instructed to return to their place of residence, consume their typical product dose, and record the amount consumed (see *Acute use cannabis dose*). Because 60 min is the average time that CBD and THC levels begin to peak in the blood after oral administration of cannabis, assessment of the acute effects of cannabis (see *Acute use change measures*) began at exactly 1-h post-use (Nadulski et al., 2005; Ohlsson et al., 1986). To account for individual differences in metabolism and sensitivity, patients were assessed a final time at 2-h post-use with the same measures.

### Measures

#### Baseline health and cannabis use

##### Demographics

Information regarding participant demographics was collected and included participant age, gender, race/ethnicity, marital status, education, and employment.

##### Health Status

The Short Form 12 Health Survey (SF-12) (Ware et al., 1996) is a reliable and valid 12-item questionnaire for pain patients (Hayes et al., 2017), designed to detect how much participants’ current health has impacted eight health-related quality-of-life domains in the past 2 weeks.

##### Negative Affect

The Depression Anxiety and Stress Scale-21 (DASS) (Chan and Lovibond, 1996; Henry and Crawford, 2005) is a 21-item measure used to assess negative affect with subscales specific to depression, anxiety, and stress (α = 0.90).

##### Cannabis Pain Expectancy

The Impact of Marijuana on Pain (IMP) assesses the benefits participants *expect* to get from cannabis prior to any cannabis use. Subjects were asked “Which of the following benefits do you expect to get from cannabis? (please select the level of change you expect)” with one category of responses being “Decreased pain”. Possible responses included “Very improved”, “Somewhat improved” “Not very improved”, or “No improvement at all”, with responses reverse coded from 3 to 0.

##### Cannabis Use Disorder (CUD)

CUD was assessed via a DSM-5 modified Marijuana Dependence Scale (Stephens et al., 2000) with 11 items (α = 0.69) which reflects total CUD symptoms reported.

##### Adverse Events

Adverse events were recorded across the study at all time points.

#### Measures of cannabis exposure

##### Frequency of Cannabis Use

The Online Timeline Followback assessment (O-TLFB) gathers information on participant substance use, including alcohol and cannabis, from the previous 14 days (Martin Willett et al., 2020). Details of cannabis use captured by the O-TLFB included form (e.g., edible, flower, concentrate) and quantity (e.g., milligrams of THC and CBD consumed, grams of flower smoked). The total number of cannabis use days reported over the 2-week study was used to measure the frequency of cannabis use over the duration of the study. Although the O-TLFB is a validated tool in measuring cannabis use frequency, the use of self-report data presents a limitation as it introduces risk of bias and error.

##### Plasma Cannabinoids

During the Baseline visit and for each of the three mobile laboratory timepoints during the Week two visit, 7 mL of whole blood was collected in EDTA-treated vacutainers by a certified phlebotomist to assess for cannabinoid exposure. Blood samples collected during the mobile laboratory visit were stored on ice until the research staff returned to the campus laboratory. Plasma supernatants were harvested and aliquoted into microcentrifuge tubes, after being centrifuged at 1,000 g for 10 min. Samples were stored at −80°C until cannabinoid analysis by the iC42 laboratory at the University of Colorado Anschutz School of Medicine. Cannabinoid analysis for THC, 11-hydroxy-THC (11-OH-THC), and CBD was completed using a validated high-performance liquid chromatography/mass-spectroscopy (HPLC-MS/MS) protocol (API550034) (Klawitter et al., 2017).

##### Acute use cannabis dose

During the acute administration mobile laboratory visit, participants recorded the amount consumed of their chosen edible product in milligrams. Acute THC and CBD doses were calculated based on the amount consumed and the previously documented labeled cannabinoid content of the product.

#### Primary outcomes measures

##### Change from pre-to post-acute cannabis use

###### Acute pain intensity

Participants’ current pain intensity was measured via a single item from the Pain Intensity Short Form 3a which asked, “What is your level of lower back pain currently?” and was rated on a scale of 0 = “no pain” to 10 = “worst imaginable pain” (Revicki et al., 2009).

###### Subjective mood

Modified tension and elation subscales from the Profile of Mood States (POMS) were used to measure acute positive and negative mood effects (Shacham, 1983; Bidwell et al., 2020). The tension subscale (negative mood) consists of 4-items on a 5-point Likert scale (nervous, anxious, unable to relax, and shaky/jittery; α = 0.77) and the elation subscale (positive mood) also consists of 4-items on a 5-point Likert scale (feeling joyful, euphoric, elated, and cheerful; α = 0.81).

###### Subjective drug effects

Subjective effects of cannabis use were assessed using two scales: the three-item Cannabis Intoxication Scale which measures mentally stoned, physically stoned (5-point scale), and feeling high (0 to 10) (α = 0.61) (Bidwell et al., 2020), and the 12-item Addiction Research Center Inventory for Marijuana Use (ARCI-M; α = 0.63) (Hill et al., 1963).

##### Change over 2 weeks of cannabis use

###### Pain intensity and interference

Participants’ average pain intensity over the past 7 days was measured using a single item from the Pain Intensity Short Form 3a which asked, “In the past 7 days, how intense was your average lower back pain?” and was rated on a scale of 0 = “no pain” to 10 = “worst imaginable pain” (Revicki et al., 2009). In addition, the Roland-Morris Disability Questionnaire (RMDQ) is a 24-item validated measure used to assess physical disability (interference) caused by low back pain (α = 0.81) (ROLAND and MORRIS, 1983).

###### Patient Global Impression of Change (PGIC) scale

The PGIC scale is a single-item, seven-point measure (Guy, 1976) with 1 = “Very much worse”, 4 = “No change”, and 7 = “Very much improved”, and is commonly used to assess participants’ perceived change in pain (Perrot and Lantéri Minet, 2019; Rampakakis et al., 2015). For the present study, this change was specific to the time between Baseline and Week two.

## Data analysis

Analyses utilized an Intent to Treat approach (Gupta, 2011) and were conducted in R (R Core Team, 2023) and Python (Version 3.8.6) (Van Rossum, 1995) using rpy2 (Version 3.5.3) (Gautier, 2008). The ggplot2 library (Version 3.3.3) (Wickham, 2016) was used for figures and the rstatix library (Version 0.7.2) (Kassambara, 2023) was used for the models.

### Plasma cannabinoid concentrations and ingested dose

To assess plasma cannabinoid concentrations over time, we employed a mixed-design analysis of variance (ANOVA) model. The factors included in the model were time (baseline, pre-use, 1-h post-use, and 2-h post-use), group (CBD-dominant, THC + CBD, THC-dominant), and the group by time interaction. As THC is rapidly metabolized into its active metabolite 11-hydroxyΔ^9^-THC (11-OH-THC), measuring this metabolite along with THC is relevant to total THC exposure in the context of short-term use (Huestis et al., 1992). Thus, for the models assessing cannabinoid concentrations after acute use, THC exposure was defined as a sum score of THC +11-OH-THC levels. The primary outcomes measured were plasma concentrations of THC +11-OH-THC and CBD, as well as the THC and CBD *doses* that participants consumed during the acute use assessments.

### Analysis of acute-use outcomes

Mixed design analysis of variance (ANOVA) was used to test the acute effects of cannabis use. Time (pre-use, 1-h post-use, and 2-h post-use), group (CBD-dominant, THC + CBD, THC-dominant), and the group-by-time interaction were included as predictors. Outcomes were pain intensity, subjective mood (POMS: tension and elation), and cannabis drug effects (Cannabis Intoxication Scale and ARCI-M). These models controlled for age and education (dummy coded as 1 = Less than bachelor’s degree, 2 = bachelor’s degree or higher). The model evaluating acute pain intensity also controlled for individual expectancies for cannabis improving pain (pain expectancy). Additional Pearson’s correlations were run to account for variations in self-reported THC and CBD *doses* that participants consumed during the acute use assessments and changes over time. For these correlations, we calculated a change score (pre-use score minus 2-h post-use score), where a positive change score indicates a decrease of the measure score over time, and a negative change score indicates an increase over time. We then correlated these change scores with THC and CBD *doses*. Similar to the ANOVAs, only the dose correlations for pain intensity included pain expectancy as a covariate. Covariates were selected based on empirical evidence supporting associations between pain and demographics of age and education (Zajacova et al., 2021). Further, controlling for expectancies is important in the context of a naturalistic design given that pain studies often report that positive expectancies are associated with better patient-reported outcomes and pain reduction (Langford et al., 2023).

### Analysis of extended (2-week) outcomes

Analyses were conducted using mixed-design analysis of variance (ANOVA) to assess changes in average pain intensity and interference (RMDQ) across the 2-week *ad libitum* use period (Baseline and Week 2). For perceived change in pain as measured by the PGIC, only the Week two timepoint was included, as this measure assessed changes in overall pain from the baseline session. Given that participants followed a naturalistic use paradigm during the 2-Week cannabis exposure period, models accounted for variation in their frequency of cannabis use during this observation period and included this as a predictor as well as time, group (CBD-dominant, THC + CBD, and THC-dominant), and all interactions (i.e., group by frequency of cannabis use, group by time, time by frequency of cannabis use, and group by frequency of cannabis use by time). As previously described, covariates were strategically selected (Zajacova et al., 2021; Langford et al., 2023); all models controlled for age, education (dummy coded as 1 = Less than bachelor’s degree, 2 = bachelor’s degree or higher), and pain expectancy (Gupta, 2011; R Core Team, 2023).

## Results

### Participants

A total of 249 participants were included in analyses, with 96 in the CBD-dominant group, 117 in the THC + CBD group, and 36 in the THC-dominant group (Table 1). There were significant differences across groups for age, race/ethnicity, and education (see Table 1). At baseline, plasma cannabinoid levels for THC, 11-OH-THC, and CBD were very low across groups, without significant differences between groups (THC: *F* (2, 200) = 0.76, *p* = .47, $\eta^{2}$ = 0.01; 11-OH-THC: all levels were zero; CBD: *F* (2, 200) = 0.27, *p* = $\eta^{2}$ = 0.003; see Supplementary Table S1).

**Table 1:** Baseline Sociodemographic, Medical Cannabis Registration, Health, and Substance Use Characteristics of Participants.

Demographics	CBD Group		THC + CBD Group		THC Group		p-value
	M	(SD)	M	(SD)	M	(SD)	
Age	50.18	(14.49)	46.06	(16.38)	36.92	(15.02)	<0.001
	n	(%)	n	(%)	n	(%)	
Gender							
Female	57	(60)	68	(58.12)	15	(41.66)	NS
Male	37	(38.95)	47	(40.17)	20	(55.55)	
Transgender/non-binary	1	(1.05)	2	(1.71)	1	(2.77)	
Race/Ethnicity							
American Indian or Alaskan Native	2	(2.08)	0	(0)	4	(11.11)	0.02
Asian	3	(3.13)	3	(2.6)	3	(8.33)	
Black or African American	4	(4.16)	2	(1.69)	3	(8.33)	
Native Hawaiian or Other Pacific Islander	1	(1.04)	1	(0.84)	0	(0)	
White	88	(91.66)	105	(88.98)	31	(86.11)	
Hispanic or Latino	3	(3.13)	11	(9.32)	3	(8.33)	
Prefer not to answer	1	(1.04)	1	(0.84)	0	(0)	
Education							
Less than Bachelor’s	26	(27.1)	29	(25)	17	(47.22)	0.01
Bachelor’s or Higher	70	(72.92)	87	(75)	19	(52.78)	
Medical Cannabis Card							
Yes	1	(96.9)	2	(2.78)	1	(1.7)	NS
No	93	(1.04)	113	(94.44)	34	(95.76)	
Not comfortable answering	2	(2.08)	2	(2.78)	1	(2.54)	
Pain Intensity	3.37	(0.92)	3.45	(0.97)	3.2	(0.93)	NS
Pain Interference	9.64	(4.98)	9.11	(4.43)	8.03	(3.58)	NS
Health Status	23.31	(3.20)	23.08	(3.3)	22.83	(2.90)	NS
Negative Affect	20.15	(17.36)	20.68	(17.75)	21.11	(14.03)	NS
Cannabis Pain Expectancy	0.89	(0.52)	0.9	(0.56)	0.94	(0.53)	NS
Cannabis Use Disorder	0.16	(0.51)	0.32	(0.98)	0.53	(0.97)	0.06
Substance Use Characteristics Past 14 days prior to Baseline							
Cannabis Use Days	0.41	(1.53)	0.6	(1.15)	0.97	(1.31)	NS
Alcohol Use Days	3.68	(3.82)	3.96	(3.89)	3.73	(3.39)	NS
Prescription Medications	54	(56.3)	67	(56.3)	21	(58.33)	NS

*Note*. *N* = 249 (n = 96 for CBD Group, n = 117 for THC + CBD Group, n = 36 for THC group). Results are reported as mean (standard deviation). All data reported in this table was collected during the Baseline visit. Health status was assessed by the SF-12. Negative affect was captured by total score on the Depression, Anxiety, and Stress Scale (DASS). Expectancies of cannabis on pain were measured on a scale of 3-0. Cannabis use disorder was assessed via the DSM-5 modified Marijuana Dependence Scale (MDS) scale. Substance Use Characteristics was measured via the Online Timeline Followback (O-TLFB) assessing the past 14 days prior to the Baseline appointment. Significant differences were found in age, as the THC group was significantly younger than the CBD group (*p* < 0.001) and the THC + CBD group (*p* = 0.007). Results showed significant differences between the groups in education ( $X^{2}$ (12, *N*=249)=21.56, *p* = 0.01) and ethnicity ( $X^{2}$ (2)=7.81, *p* = 0.02). Of the 142 participants who endorsed prescription medication across the sample, 17 (6.83%) endorsed opioids, 43 (17.37% endorsed anti-depressants, 12 (4.82%) endorsed benzodiazepines, 8 (3.21%) endorsed beta blockers, 2 (0.8%) endorsed buspirone, 10 (4.02%) endorsed sleep medication, 13 (5.22%) endorsed migraine medication, 24 (9.64%) endorsed muscle relaxants, 11 (4.42%) endorsed non-steroidal anti-inflammatory medications, 19 (7.63%) endorsed nerve pain medication, 12 (4.82%) endorsed attention-deficit/hyperactivity disorder (ADHD) medication, and 66 (26.51%) endorsed other medications [e.g., Suboxone, Quetiapine (Seroquel)].

### Adverse Events

No serious adverse events were reported during the study. One participant fainted during the blood draw at baseline. Fifteen tachycardia events where participant heart rate exceeded 100 bpm occurred after use of their cannabis product (5 in the CBD group, 8 in the THC + CBD, and 2 in the THC group) and these did not significantly differ by product group. No other adverse events were reported during the study.

### Acute responses to cannabis

#### Plasma cannabinoid concentrations and ingested dose

**FIGURE 3:**
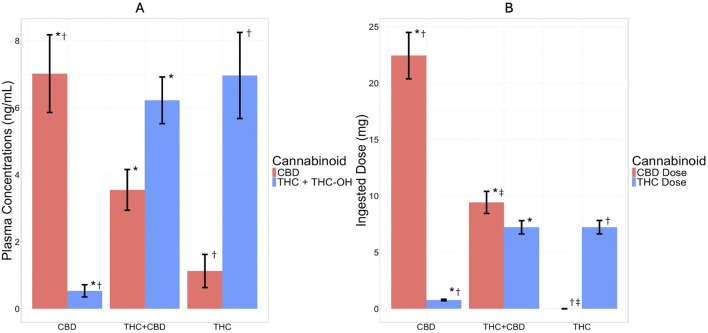
Plasma Concentrations (ng/mL) and Ingested Dose (mg) by Cannabis Product Group (CBD, THC + CBD, and THC) During Acute Mobile Laboratory Session. The bar graph depicts cannabidiol (CBD) plasma concentration and dose in red and tetrahydrocannabinol (THC) plus the blood THC metabolite 11-Hydroxy-Δ^9^-THC (11-OH-THC) plasma concentration and THC dose in blue for each cannabis product group. **(A)** Plasma concentration (ng/mL) of CBD and THC + THC-OH by product group at 1-h post-use and **(B)** CBD and THC ingested doses (mg) by product group during the acute use timepoint. Bars are graphed as ± mean (standard deviation). * = significant (*p* < 0.001) group difference between the CBD and THC + CBD groups, † = significant (*p* < 0.001) group difference between the CBD and THC groups, ‡ = significant (*p* < 0.01) group difference between the THC + CBD and THC groups. For cannabinoid exposure as measured by plasma concentrations (ng/mL), it was found that after acute cannabis use there was an expected and significant group by time interaction such that THC exposure (THC +11-OH-THC) was greater among the THC and THC + CBD groups compared to CBD, and CBD was greater within the CBD group compared to the THC and THC + CBD groups. In regard to ingested dose, these results also followed expected patterns by group such that the THC ingested dose was lower for the CBD group compared to both the THC + CBD and THC groups; the CBD ingested dose was greater for the CBD group compared to the THC + CBD and THC groups; and the CBD ingested dose was greater for the THC + CBD group compared to the THC group.

In order to verify cannabinoid exposure across product groups, models were run examining plasma cannabinoid concentrations across time, group, and group by time. There was an expected and significant group by time interaction (THC +11-OH-THC plasma concentrations: *F* (1, 759) = 36.3, *p* <. 001, $\eta^{2}$ = 0.05; CBD plasma concentrations: *F* (1, 759) = 6.79, *p* = $\eta^{2}$ = 0.01). Post hoc analyses showed that there were no group differences in plasma concentrations of THC, 11-OH-THC, or CBD at the baseline session or before use at the acute session (*p*s > .90). However, there were expected differences between groups at both post-use timepoints where THC exposure (THC +11-OH-THC) was greater among the THC and THC + CBD groups compared to CBD, and CBD was greater within the CBD group compared to the THC and THC + CBD groups (see Figure 3; Supplementary Table S1 for a complete reporting across timepoints). Similarly, there was a significant main effect of group on the THC and CBD doses (mg) ingested during the acute use session (group effect on THC dose ingested: *F* (2, 226) = 77.9, $\eta^{2}$ <.001, = 0.26; group effect on CBD dose ingested: *F* (2, 226) = 76.4, $\eta^{2\;}$ <. 001, = 0.25; Table 2 and Figure 3B). Post hoc analysis showed the THC ingested dose was lower for the CBD group compared to both the THC + CBD and THC groups; the CBD ingested dose was greater for the CBD group compared to the THC + CBD and THC groups; and the CBD ingested dose was greater for the THC + CBD group compared to the THC group (See Figure 3). See Supplementary Figure S1 for the full range of THC and CBD doses reported across the study group.

**Table 2:** Acute Use Cannabis Dose (mg) of THC and CBD.

	CBD Group	THC + CBD Group	THC Group
THC (mg)	0.78^a^ ^,^ ^b^	8.07^a^	7.23^b^
	(0.65)	(11.04)	(3.4)
CBD (mg)	22.57^a^ ^,^ ^b^	10.30^a^ ^,^ ^c^	0.01^b^ ^,^ ^c^
	(19.55)	(13.76)	(0.05)

*Note*. *N* = 249 (n = 96 for CBD Group, n = 117 for THC + CBD Group, n = 36 for THC group). Results are reported as mean (standard deviation). Calculated doses for THC and CBD are reported in milligrams (mg) and are based on cannabinoid content present on the participant’s submitted product label and reported amount consumed during the acute use session.

^a^ Significant (*p* < 001) group difference between the CBD and THC + CBD groups.

^b^ Significant (*p* < 001) group difference between the CBD and THC groups.

^c^ Significant (*p* < 01) group difference between the THC + CBD and THC groups.

#### Pain intensity

**FIGURE 4:**
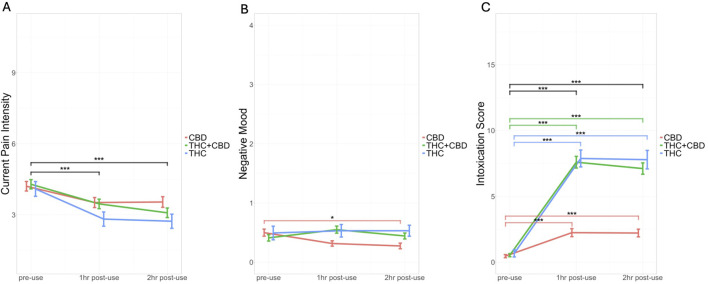
Changes in Pain Intensity, Negative Mood, and Intoxication Score by Cannabis Product Group (CBD, THC + CBD, and THC) During Acute Mobile Laboratory Session. The colored line graphs represent cannabis product group including CBD (red), THC + CBD (green), and THC (blue) and changes over time during the acute use session [before cannabis use (pre-use), 1-h post-use, and 2-h post-use]. **(A)** Self-reported current pain intensity (Pain Intensity Short Form 3a) by product group by time **(B)** self-reported negative mood [Profile of Mood States (POMS)] by product group by time **(C)** self-reported drug effects [Cannabis Intoxication Scale (average of feeling high, mentally stoned, and physically stoned)] by product group by time. Bars are graphed as ± mean (standard deviation). Significant differences between timepoints and within groups are represented by colored lines corresponding to the color of the group and significant effects of time are represented by black lines. *** = *p* < 0.001, **p < 0.01, and *p < 0.05. For current pain intensity, all groups reported experiencing lower pain intensity over the course of the acute use session. For negative mood, there was a group by time interaction such that the CBD group experienced a decrease in tension over the course of the acute use session. Finally, for cannabis intoxication, there was a group by time interaction such that there were significant increases in intoxication scores from pre-use to both 1-h and 2-h post-use across all groups, however this increase was steeper for the THC and THC + CBD groups compared to the CBD group.

The model examining acute pain intensity with product group as a predictor indicated a small significant effect of time (*F* (1, 682) = 30.00, *p* < .001, $\eta^{2\;}$ = 0.04) and a main effect of age (*F* (1, 682) = 4.45, *p* = .04, $\eta^{2}$ = 0.01), where pain intensity decreased after cannabis administration and older patients reported worse pain. There was no main effect of group or significant interaction between group and time (*p*s > .05; Figure 4A). When addressing the influence of dose of cannabis consumed during the acute use session, results demonstrated a significant positive correlation between change in pain intensity and THC dose (*r* (227) = 0.14, *p* = .03), such that higher THC doses were associated with larger decreases in pain. There was no significant correlation between change in pain intensity and CBD dose (*r* (227) = 0.05, *p* = .14).

#### Negative mood (POMS): Tension

For negative mood, there were main effects of age (*F* (1, 684) = 20.90, *p* < .001, $\eta^{2}$ = 0.03) and education (*F* (1, 684) = 5.18, *p* = .02, $\eta^{2}$ = 0.01), such that younger people and more educated people reported higher levels of negative mood. There was also a significant group by time interaction (*F* (1, 684) = 5.02, *p* = .03, ${\;\eta }^{2}$ = 0.01). Specifically, the CBD-dominant group exhibited a decrease in tension (difference = 0.23, SE = 0.08, *p* = .01), whereas the THC-dominant and THC + CBD groups showed no change (*p*s > .05; Figure 4B). The correlations addressing cannabinoid dose further identified a significant negative correlation between the tension change score and THC dose (*r* (228) = −0.21, *p* = .001), suggesting that regardless of product grouping higher THC doses were associated with increases in tension. There were no significant correlations between change in negative mood and CBD dose (*r* (228) = 0.03, *p* = .66).

#### Positive mood (POMS): Elation

The model examining elation resulted in a main effect of time, in which elation decreased significantly over time (*F* (1, 684) = 4.9, *p* = .03, $\eta^{2}$ = 0.01) and no other significant effects. A similar trend was found when looking at the dose correlations, where the change in elation was significantly negatively correlated with THC dose (*r* (228) = −0.15, *p* = .03), suggesting that lower THC doses resulted in greater decreases in elation. There was no significant correlation between change in elation and CBD dose (*r* (228) = 0.11, *p* = .09).

#### Cannabis drug effects

The intoxication score reflected significant age, group, time, and group-by-time interaction effects (age: *F* (1, 684) = 12.80, *p* < .001, $\eta^{2}$ = 0.02; group: *F* (1, 684) = 103.00, *p* < .001, $\eta^{2}$ = 0.13; time: *F* (1, 684) = 214.00, *p* < .001, $\eta^{2\;}$ = 0.24; group-by-time interaction: *F* (1, 684) = 42.90, *p* < .001, $\eta^{2}$ = 0.06). Simple effects tests show significant increases in intoxication scores from pre-use to both 1-h and 2-h post-use across all groups, but this increase was steeper for the THC (difference = 7.21, SE = 0.78, *p* < .001) and THC + CBD groups (difference = 7.12, SE = 0.43, *p* < .001) compared to the CBD group (difference = 1.74, SE = 0.47, *p* < .001) (Figure 4C). There was a significant negative correlation between intoxication change score and THC dose (*r* (228) = −0.37, *p* < .001). With higher THC doses being associated with greater intoxication. Further, there was a significant positive correlation between intoxication change score and CBD dose (r (228) = 0.24, *p* = .003). Suggesting decreases in intoxication as CBD dose increased. Similarly, the ARCI-M scores demonstrated significant effects of age, group, time, and group-by-time interaction (age: *F* (1, 681) = 16.90, *p* < .001, $\eta^{2}$ = 0.02; group: *F* (1, 681) = 61.00, *p* < .001, $\eta^{2}$ = 0.08; time: *F* (1, 681) = 165.00, *p* < .001, $\eta^{2}$ = 0.20; group-by-time interaction: *F* (1, 681) = 21.00, *p* < .001, $\eta^{2}$ = 0.03). Simple effects tests show a similar increase in perceived drug effects from pre-use to 1-h and 2-h post-use for all groups, with a steeper increase for the THC (difference = 3.49, SE = 0.58, *p* < .001) and THC + CBD groups (difference = 4.23, SE = 0.32, *p* < .001) compared to the CBD group (difference = 1.28, SE = 0.35, *p* < .001). There was a significant negative correlation between drug effect change score and THC dose (*r* (227) = −0.26, *p* < .001), suggesting that as THC dose increased, the change in drug effects also increased. Conversely, a significant positive correlation was found between drug effect change scores and CBD dose (*r* (227) = 0.20, *p* = .003). These findings imply that greater doses of CBD were associated with lower changes in subjective drug effects.

### Pain over two weeks of cannabis use

For the 2-week exposure period, models for pain intensity and pain interference (see Supplementary Table S2) resulted in significant main effects of time indicating an overall reduction of pain levels and interference. There were no group-by-time interactions on either outcome, suggesting that changes in pain intensity and interference consistently decreased across groups over the 2-week period. We note that the pain intensity model showed a trend for a group-by-frequency of use interaction (*p* = .07), suggesting that at average and higher frequencies of use, the CBD group had the lowest levels of pain intensity (see Supplementary Table S2). In addition, across the 2-week study, the CBD-dominant group reported significantly lower levels of average pain intensity compared to the THC + CBD group (difference = −0.57, SE = 0.17, *p* = .003). There were also main effects of age, education, pain expectancy, and group on pain intensity. Older individuals tended to report higher pain intensity, while those with higher education levels and lower pain expectancy (meaning they anticipated less pain reduction with cannabis use) reported lower pain intensity. Finally, in the model assessing perceived change in pain using PGIC, there was a main effect of frequency of study product use (see Supplementary Table S2) showing that perceived pain improvement increased as frequency of use increased.

## Discussion

This is a highly novel human clinical trial on the association of naturalistic administration of legal market edible cannabis products on chronic pain, intoxication, and mood. The study aimed to evaluate the acute and extended effects of legal market edible cannabis use on pain and other relevant outcomes. In addition, these findings are reported in a sample of participants with chronic low back pain providing data on naturalistic use patterns and safety in individuals newly initiating cannabis use for their back pain. Along these lines, participants reported *ad libitum* use of range of products and doses. Further it was found that initiating cannabis use resulted in minimal adverse events, which did not differ across product groups. The study was well-balanced in gender and more diverse in age than the majority of cannabis use studies (Barkholtz and Bates, 2023; Schlienz et al., 2017; Martin-Willett and Bidwell, 2021).

During the acute cannabis administration period, pain intensity following edible cannabis use decreased over time across all three broadly defined product groups. Further analyses revealed an association between decreases in pain intensity and higher doses of THC. With significantly greater decreases in pain intensity 1- and 2- hours post-use. These findings, consistent with our primary hypothesis, suggest that higher THC doses were more effective for acute relief of chronic pain, but that CBD, at these naturalistic doses, did not have an additive effect. Previous literature supports our findings on the effects of THC for acute pain relief (Hill, 2015; Whiting et al., 2015b). However, there is limited research suggesting that CBD also has acute analgesic effects (Gulbransen et al., 2020; Palmieri et al., 2017), which was not found in the present study. Continued research, particularly on recreational and medically accessible cannabis products, is greatly needed to improve our understanding of the effectiveness of these products and their cannabinoid profiles for pain relief.

As expected, higher doses of THC increased the intoxication and drug effects reported across individuals (Ashton, 2001)and CBD doses did not significantly associate with intoxication effects over time, supporting the non-intoxicating properties of CBD as seen in previous literature (Solowij et al., 2019; Kicman and Toczek, 2020). Interestingly, in the group analysis, both the THC-dominant and THC + CBD edible groups showed similar levels of subjective drug effects, suggesting that including CBD in the edible did not alter THC-induced subjective drug effects in the current study (Wall et al., 2022; Gautier, 2008). Moreover, significant differences between product group and mood outcomes were seen as the CBD-dominant group reported reduced tension as compared to the THC-dominant and THC + CBD groups. Further, when evaluating cannabinoid dosage and mood outcomes across all three of the product groups, higher doses of THC were associated with a tension increase. These results are in agreement with previous research supporting that CBD is an effective anxiolytic and can reduce negative affect (Zuardi et al., 2017; Crippa et al., 2011), whereas THC can be anxiogenic in a dose-dependent manner (Sharpe et al., 2020; Rosário et al., 2024; Lichenstein, 2022). Thus, any benefits of THC on short-term pain relief should be balanced with the potential impact on negative mood and intoxication that was also associated with THC in our study.

Broadly, over the extended 2-week administration period, there were no significant group-by-time interactions which suggests that changes in overall pain levels consistently decreased across individuals who initiated cannabis regardless of the product they selected. Yet, some trends in our data suggested that participants using a CBD-dominant product, particularly those who used it more frequently, showed lower pain levels over the 2-week period. These findings are somewhat equivocal and beg the question of whether CBD, especially in the doses typically consumed via legal market edible forms of cannabis, has a role in longer-term relief from chronic back pain. However, other data support the trends shown in our study. For example, in a prospective study evaluating the efficacy of CBD hemp extract (∼30 mg daily) on chronic pain patients over an extended 8-week period, it was found that the use of CBD significantly reduced pain intensity and interference (Capano et al., 2020). Thus, the 2-week extended findings combined with support from prior research suggest that CBD, particularly under conditions of steady use and higher doses, should continue to be considered for its potential in long-term pain relief. Clearly, more research is warranted as there currently exists limited clinical trial data evaluating CBD-dominant products compared to THC-dominant products for chronic pain relief, and a majority of studies have evaluated CBD in combination with THC, such as nabiximols, for pain management (Arkell et al., 2022; Boyaji et al., 2020; Serpell et al., 2014; Überall, 2020; Urits et al., 2020).

### Strengths and limitations

Several methodological strengths and limitations should be considered in the interpretation of our results. This study examined a community sample who used legal market cannabis *ad libitum*, allowing for a naturalistic observation of how participants may choose to use cannabis in their daily lives to reduce their chronic pain. Given individual differences in cannabis metabolism and tolerance, our *ad libitum* dosing procedure provides important information that is consistent with real-world use. Thus, external validity is a notable strength of these findings as the ratios of THC to CBD in legal market products are more accurate to typical medical and recreational use than what is available to researchers for laboratory-based studies (Vergara et al., 2017). This approach, however, lacks a placebo- and dose control, thus limiting the inferences that can be made about cannabinoid use and dosing across study participants. In addition to these limitations, much, but not all, of the participant health data is based on self-report. For example, to assess cannabis use and exposure, we utilized the O-TLFB, participant reported dose in milligrams, and plasma cannabinoid levels (Supplementary Table S1; Supplementary Figure S1). Future analyses should include evaluation of the associations of cannabis use and objective pain and inflammation biomarkers. Further, it is important to note that there is variation across the different forms of legal market edible cannabis in terms of additives and minor cannabinoids that was not accounted for in the current study. Finally, while research staff were blind to the cannabinoid content of the assigned product, participants were not blinded, thus their expectancies regarding the effectiveness of THC and CBD for pain mediation, while controlled statistically in the analysis, may have impacted the results.

## Conclusion

In this naturalistic observational study, it was found that the use of edible cannabinoid products significantly reduced chronic pain in extended and acute use models. More specifically, THC dose was associated with the greatest decrease in pain during the acute use session. Further, there was signal that more frequent use of a CBD-dominant product may provide stronger relief over a 2-week *ad libitum* use period. These results indicate that edible cannabis may be a safe and suitable alternative pain therapy for those looking to substitute more traditional pain medications. With the rapidly evolving cannabis landscape, these data spur future research into differential short- and long-term effects of cannabinoid products that directly compares various doses of THC and CBD. Future studies should seek to establish the lowest effective dose in order to prevent over consumption in individuals turning to this form of therapy and better inform public health policy and cannabis regulation. Continued research on the effectiveness of varying cannabis products for chronic pain is critical to expand our knowledge base on the potential therapeutic value and side effects of short- and long-term cannabinoid use.

## Data Availability

The original contributions presented in the study are included in the article/Supplementary Material, further inquiries can be directed to the corresponding author.
