# Supplementary figures and images for: Edible cannabis for chronic low back pain: associations with pain, mood, and intoxication
Source: Front Pharmacol. 2024 Sep 24;15:1464005. doi: 10.3389/fphar.2024.1464005 (PMC11458467; doi:10.3389/fphar.2024.1464005)

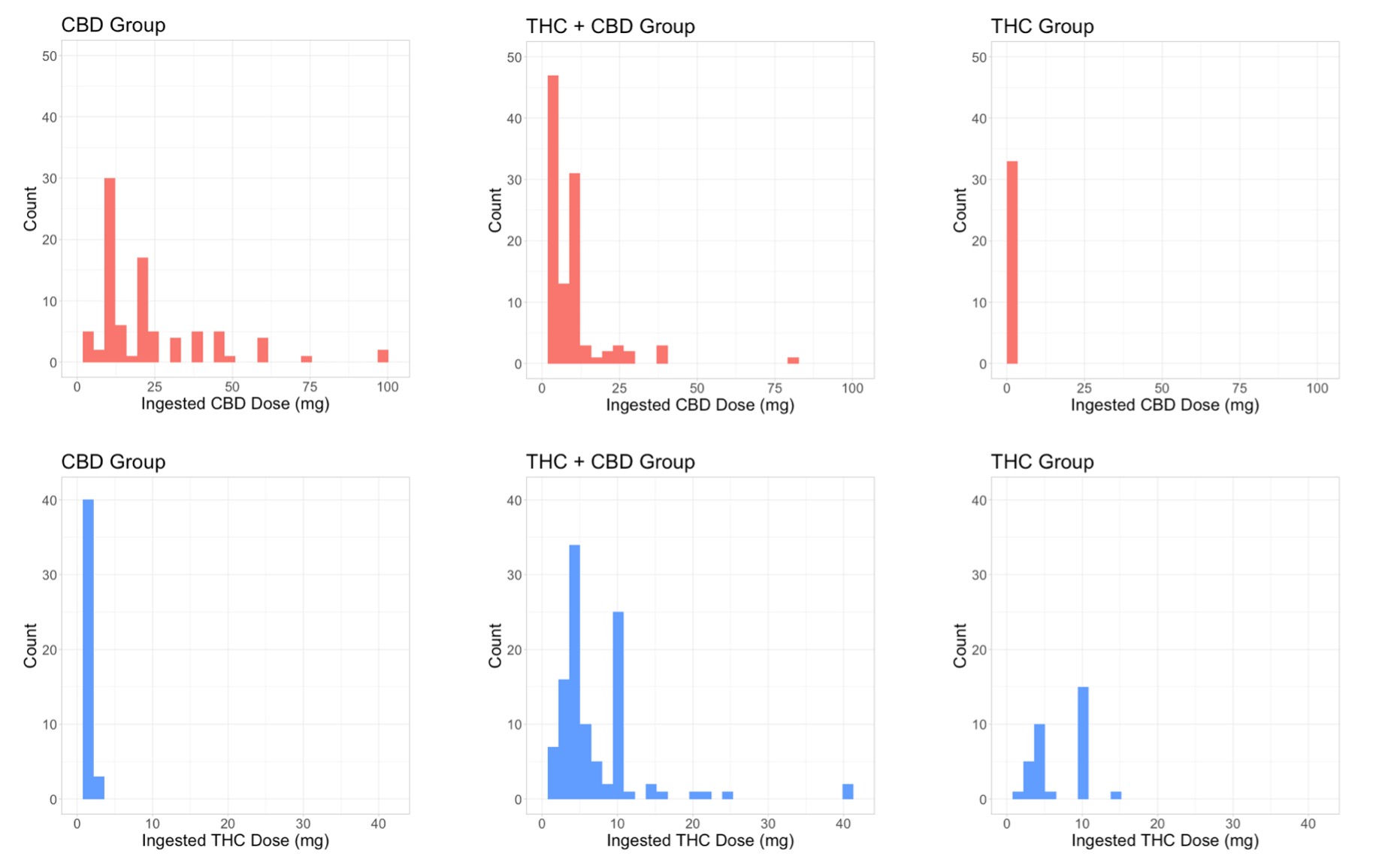

Supplement: Supplementary file 1 [file Image1.JPEG]
