# Supplementary material for: Edible cannabis for chronic low back pain: associations with pain, mood, and intoxication
Source: Front Pharmacol. 2024 Sep 24;15:1464005. doi: 10.3389/fphar.2024.1464005 (PMC11458467; doi:10.3389/fphar.2024.1464005)
Supplement: Supplementary file 2 [file DataSheet1.pdf]

Supplementary Materials

Supplementary Table 1. Plasma Cannabinoid Concentration by Timepoint

	CBD Group			THC + CBD Group			THC Group		
	THC (ng/mL)	11-OH-THC (ng/mL)	CBD (ng/mL)	THC (ng/mL)	11-OH-THC (ng/mL)	CBD (ng/mL)	THC (ng/mL)	11-OH-THC (ng/mL)	CBD (ng/mL)
Baseline	0.01 (0.06)	0.00 (0.00)	0.02 (0.12)	0.02 (0.2)	0.00 (0.00)	0.02 (0.17)	0.04 (0.15)	0.00 (0.00)	0.05 (0.24)
Pre-use	0.02 (0.16)	0.05 (0.34)	**0.74*** (2.01)	0.09 (0.46)	0.07 (0.41)	**0.21*** (0.47)	0.19 (0.51)	0.11 (0.52)	0.12 (0.39)
1-hour post-use	**0.25***† (0.55)	**0.28***† (1.19)	**7.02***† (10.49)	**3.43*** (4.30)	**2.79*** (2.86)	**3.55*** (5.84)	**4.21**† (4.05)	**2.75**† (2.56)	**1.13**† (2.42)
2-hour post-use	**0.13***† (0.29)	**0.07***† (0.25)	5.23 (7.09)	**2.03*** (2.52)	**1.94*** (2.07)	3.14 (6.55)	**2.16**† (2.07)	**1.60**† (1.60)	3.37 (12.30)

Note. Plasma cannabinoid concentrations are reported in nanograms per milliliter (ng/mL) at baseline, pre-use, 1-hour post-use, and 2-hours, post-use for each product group (CBD, THC + CBD, and THC). Data are reported as mean (standard deviation). * = significant ($p<.05$) group difference between the CBD and THC + CBD groups, † = significant ($p<.05$) group difference between the CBD and THC groups, ‡ = significant ($p<.05$) group difference between the THC + CBD and THC groups.

Supplementary Table 2. 2 Week Average Pain Intensity and Pain Interference ANOVA Models

	Average Pain Intensity			Pain Interference			Perceived Change in Pain		
Predictors	*F*	Part η^2 [95%CI]	*p*-value	*F*	Part η^2 [95%CI]	*p*-value	*F*	Part η^2 [95%CI]	*p*-value
**Extended models**									
Age	6.20	0.02 [0.00,0.05]	.01	15.48	0.04 [0.01,0.08]	<.001	0.78	0.00 [0.00,0.03]	.38
Education	10.72	0.03 [0.01, 0.06]	.001	0.40	0.00 [0.00, 0.02]	.52	0.21	0.00 [0.00,0.03]	.65
Pain Expectancy	7.03	0.02 [0.00, 0.05]	<.01	3.23	0.01 [0.00, 0.03]	.07	1.57	0.01 [0.00,0.04]	.21
Group	5.74	0.01 [0.00, 0.04]	.02	0.62	0.01 [0.00, 0.03]	.43	1.76	0.01 [0.00,0.05]	.19
Time	18.14	0.04 [0.01, 0.08]	<.001	9.20	0.02 [0.00, 0.05]	<.01	-	-	-
Frequency of Use	2.21	0.00 [0.00, 0.02]	.14	0.07	0.00 [0.00, 0.02]	.79	5.21	0.02 [0.00,0.08]	.02
Group x Time	0.25	0.00 [0.00, 0.01]	.62	0.11	0.00[0.00, 0.01]	.74	-	-	-
Group x Frequency of Use	3.32	0.01 [0.00, 0.03]	.07	0.37	0.00 [0.00, 0.01]	.55	1.11	0.00 [0.00,0.04]	.29
Frequency of Use x Time	1.13	0.00 [0.00, 0.02]	.29	0.41	0.00 [0.00, 0.01]	.52	-	-	-
Group x Frequency of Use x Time	0.03	0.00 [0.00, 0.01]	.86	0.01	0.00 [0.00, 0.00]	.92	-	-	-

Note. Supplementary Table 2 provides the full ANOVA results between average pain intensity, pain interference, and perceived change in pain over the 2 week period. The perceived change in pain is only collected at one timepoint, therefore no effects of time or its interactions are included in this model.

Supplementary Figure 1.

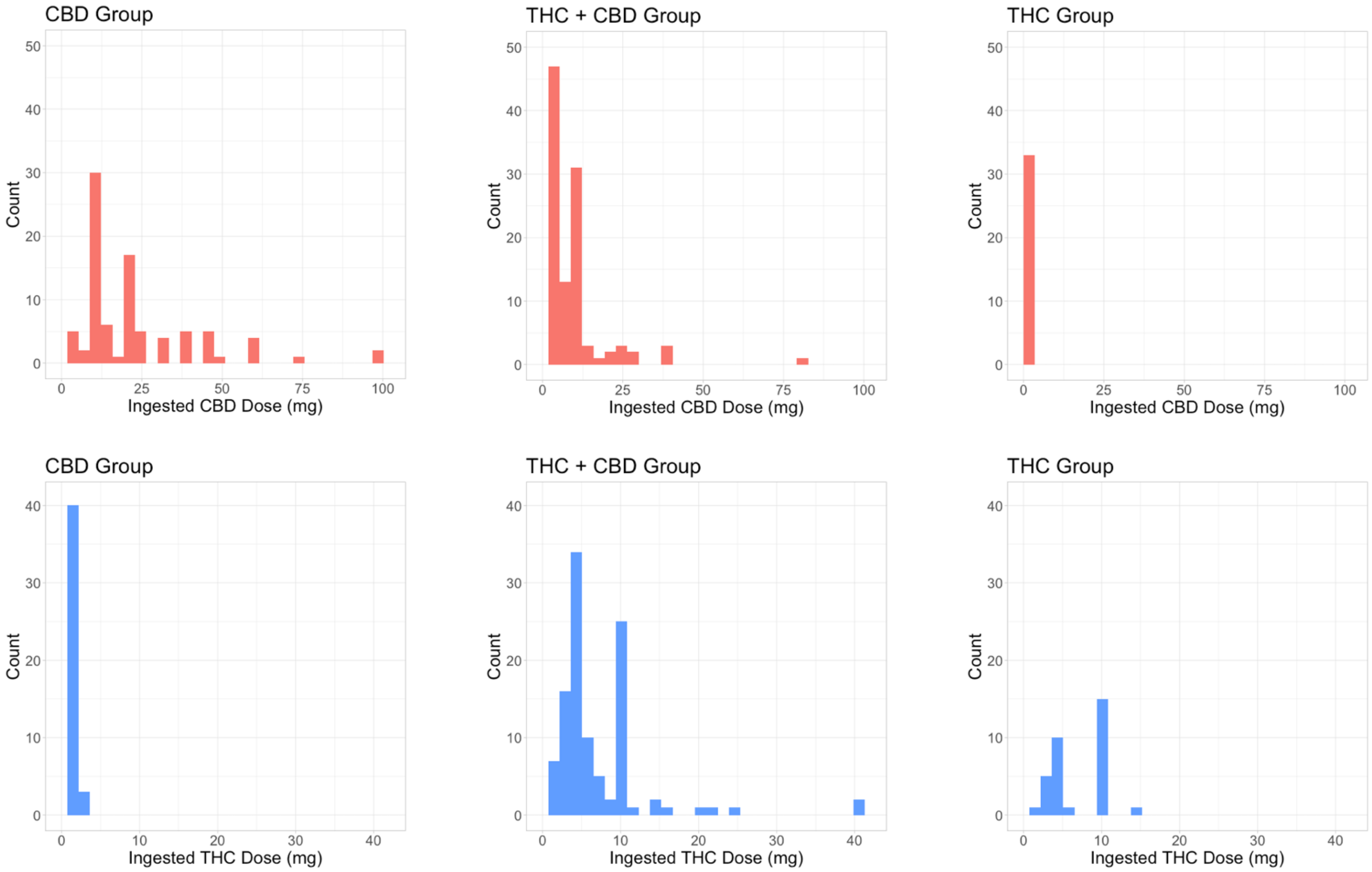

Note. Supplementary Figure 1 includes six bar graphs which depicts the ingested dose (mg) of CBD (red) and THC (blue) for each cannabis product group (CBD, THC + CBD, and THC) during the acute-use time point which took place in our mobile pharmacology laboratory.
